# Moderate methodological quality in the 50 most cited studies on meniscus root tears: A bibliometric study and methodological quality analysis

**DOI:** 10.1002/jeo2.70318

**Published:** 2025-07-02

**Authors:** Romed P. Vieider, Jonathan D. Hughes, Bryson P. Lesniak, Matthew Kolevar, Anja M. Wackerle, Karina Dias, Camila Grandberg, Volker Musahl

**Affiliations:** ^1^ Department of Orthopaedic Surgery, UPMC Freddie Fu Sports Medicine Center University of Pittsburgh Medical Center Pittsburgh Pennsylvania USA; ^2^ Department of Sports Orthopaedics Technical University of Munich Munich Germany

**Keywords:** arthroscopy, knee, meniscus repair, meniscus root tear

## Abstract

**Purpose:**

The aim of this study was to analyze the 50 most cited studies on meniscus root tears, analyzing their bibliographic parameters and methodological quality. The hypothesis of this study was that citations would not correlate with study quality.

**Methods:**

A literature search on Web of Science was performed to determine the 50 most cited studies on the topic of 'meniscus root' in human orthopaedics between 1 January 1990 and 9 December 2024. Bibliographic parameters and Modified Coleman Methodological Score (MCMS; 0–100 points), Methodological Index for Non‐Randomised Studies (MINORS 0–24 points), Biomechanics Objective Basic Science Quality Assessment Tool (BOBQAT; 0–100 points) and Radiologic Methodology and Quality Scale (MQCSRE; 0–30 points) were evaluated. The Mann–Whitney *U* test and Kruskal–Wallis test were used to compare groups. Linear regression analysis evaluated whether total citations correlate with study quality scores.

**Results:**

The 50 most cited studies on meniscus root tears averaged 165 ± 100 (range 92–721) citations per article, with a mean citation density of 15.3 ± 8.1 per year (range 6.4–45.1). The *American Journal of Sports Medicine* published 18 studies (36%), and 25 studies (50%) originated from institutions in the United States. Clinical (*n* = 17, 34%) and biomechanical (*n* = 13, 26%) studies were most common. Mean quality scores were: MCMS 64.9 ± 7.0, MINORS 14.9 ± 4.7, BOBQAT 80.6 ± 6.9, and RQMS 20.2 ± 1.5. Mean citation densities were higher in biomechanical studies (17.0 ± 10.1) and clinical studies (15.0 ± 5.8) than in radiological studies (9.6 ± 2.5; *p* = 0.022). Studies published in the United States had higher mean citation densities (18.7 ± 9.6) than those from Korea (12.1 ± 3.6; *p* < .001) or Germany (10.9 ± 3.9; *p* < 0.026). Citations or citation densities were not correlated with study quality (*p* = 0.282).

**Conclusion:**

The methodological quality of the 50 most cited studies on meniscus root tears was moderate, and the number of citations did not correlate with their quality scores. This list can serve as a valuable reference for orthopaedic surgeons seeking to explore the literature on meniscus root tears.

**Level of Evidence:**

Level V, cross‐sectional study.

**Abbreviations**
Am J Sports Med: american journal of sports medicine
J Bone Joint Surg Am: journal of bone and joint surgery american volume
J Bone Joint Surg Br: journal of bone and joint surgery british volume
Knee Surg Sports Traumatol Arthrosc: knee surgery, sports traumatology, arthroscopy
LOE: level of evidence
MCMS: modified coleman methodological score
MINORS: methodological index for non‐randomised studies
MQCSRE: methodological quality for clinical studies of radiologic examinations
USA: United States of America
WOS: web of science

## INTRODUCTION

Meniscus root tears represent a common entity in orthopaedic practice. A tear of the meniscus root is equivalent to a total loss of meniscal function and leads to elevated tibiofemoral contact forces in the medial or lateral knee compartment, resulting in the progression of early osteoarthritis [4, 31]. Based on these biomechanical principles and association with advances in minimally invasive surgery, various techniques for restoring the integrity of the meniscus root have been suggested [2, 17, 20, 22]. These techniques demonstrate favourable clinical and functional outcomes, effectively preventing the progression of osteoarthritis following surgical treatment of meniscus root tears [8, 14].

There is significant interest in orthopaedic literature regarding the meniscus root. To date, the search for the term 'meniscus root' on the National Library of Medicines databank PubMed yields 1117 articles published from 1979 to 2025, with more than half of these articles (*n* = 651) published in the last 5 years. This abundance of recent studies may pose challenges in establishing a comprehensive foundation on the topic. Review articles and meta‐analyses represent essential tools for obtaining an overview of a specific topic or summarising cohorts and their outcomes in an area of interest. However, information on the impact in the literature of the included studies is not provided. Bibliometric analyses provide a useful tool to obtain an initial overview of the most impactful studies in almost any medical specialty [3, 11, 12, 15, 16, 28, 29, 33, 38]. Citation counts and derived citation indices are valuable tools for assessing the impact of journals, authors, and articles to identify the trends, centres of competence, types of research articles, and possible controversies within a certain field of research. However, their relationship to a study's methodological rigour remains unclear. High citations may reflect recognition without ensuring scientific validity, while high‐quality studies in niche fields, like in the field of meniscus root tears, might be under‐cited. One way to address this issue is to obtain study quality scores. These scores assess the quality of research in a clear and simple way and allow an objective evaluation of studies, regardless of how well‐known they are in the scientific community [1, 7, 23]. This study aimed to systematically assess the 50 most cited studies on meniscus root tears, focusing on their bibliometric parameters and their methodological study quality. The hypothesis of this study was that citations would not correlate with study quality.

## METHODS

### Literature search

A search of Thompson and Reuters Web of Science Core Collection database was performed by R.P.V. and A.M.W in December 2024. As of December 2024, the WoS lists over 92 million records across 254 disciplines. All articles published between 1 January 1990 and 9 December 2024 with available abstracts were included. The search term 'Meniscus Root OR Meniscal Root' was used in the search category 'topic' to identify all articles that address this subject. Subsequently, results were filtered by their number of citations and ranked in descending order. The complete record of the first 1160 studies and related characteristics such as author name, publication year, journal, title, abstract, total citations (all databases), and language was exported into a data sheet. A comparison of search results with Elsevier's Scopus database revealed perfect concordance for the term 'meniscus root', therefore an additional extraction was not performed. All analyses of the articles were performed in December 2024.

### Inclusion and exclusion criteria

The following inclusion criteria were applied to the exported 1160 studies. English articles published in international peer‐reviewed journals between 1 January 1990 and 9 December 2024, with the term “meniscus root” in their title, were included. In addition, the abstracts of all articles were screened to identify studies that did not have the term in the title but whose main topic or outcome addressed meniscus root tears. Subsequently, these studies were also included.

**Figure 1:**
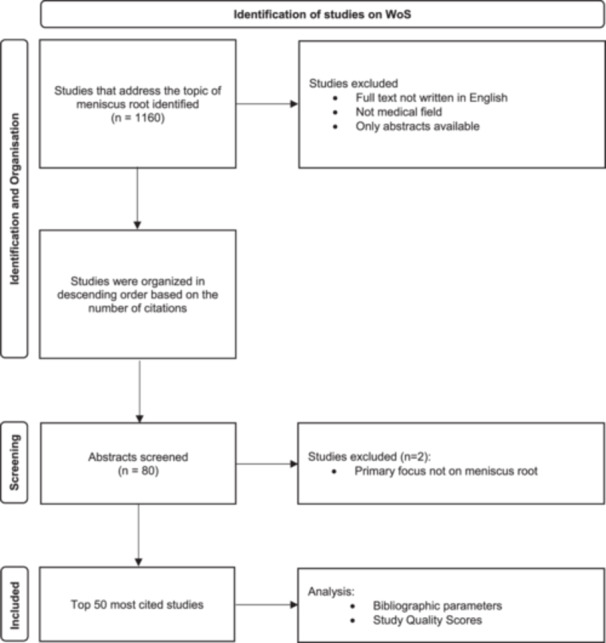
PRISMA flowchart of the study‐selection process of the 50 most cited studies on the topic 'meniscus root'. Number of studies (*n*), Web of Science (WoS).

Articles that were not published in the field of medicine, whose main topic did not relate to meniscus root tears or were not written in English were excluded (Figure 1).

### Data assessment

The 50 most cited studies regarding the topic meniscus root included in this review were evaluated by R.P.V. and A.M.W Bibliographic parameters such as first author, journal, journal impact factor (JIF) for 2024, total citation count, study type, country of origin, and year of publication were recorded. For better comparability, the citation density (total citations divided by the years since publication) was calculated for every study. Studies were then assigned to a Level of evidence (LOE) according to the Journal of Bone and Joint Surgery (JBJS) [30].

Furthermore, study quality assessment was determined by the following scores: The Modified Coleman methodological Score (MCMS) and the Methodological Index for Non‐Randomised Studies (MINORS). The MCMS (0–100 points) is a reliable tool for evaluating clinical studies and focusing on the cohort size, follow‐up rate, diagnostic workup, and rehab protocols [13]. The MINORS (0–24 points) was originally designed to quantify the methodological quality of observational studies predominantly used in surgical fields [32]. The effects of meniscus root tears have been investigated in several highly cited biomechanical studies. Furthermore, the recently published Biomechanics Objective Basic Science Quality Assessment Tool (BOBQAT) was utilised to evaluate the quality of these studies (0–100 points) [21]. Since the diagnostic workup of meniscus root tears relies on radiological methods, the scale of methodological quality for clinical studies of radiologic examinations (MQCSRE) was used to assess the quality for this study‐type (0–30 points) [6]. Anatomical studies, reviews, and meta‐analyses were not assessed for quality. Furthermore, the previously published definitions for interpreting study quality scores are summarised in Table 1 [13, 21, 32].

**Table 1:** Study quality score thresholds are displayed by the scoring system for categorising study quality as 'Excellent', 'Good', 'Moderate', 'Fair', or 'Poor' based on different study quality scores.

Score system	Excellent	Good	Moderate	Fair	Poor
MCMS (points)	85–100	70–84	55–69	Not specified	<55
MINORS (points)	24	16	Not specified	Not specified	Not specified
BOBQAT (points)	90–100	80–89	70–79	60–69	<60
MQRSE (points)	Not specified	Not specified	Not specified	Not specified	Not specified

*Note*: No details for any thresholds are given for the Methodological Quality for Clinical Studies of Radiologic Examinations (RQMS) [6].

Abbreviations: BOBQAT, biomechanics objective basic science quality assessment tool; MCMS, modified coleman methodological score; MINORS, methodological index for non‐randomised studies.

### Statistical analysis

Descriptive statistics were calculated using SPSS (IBM, Version 27) and presented in tables and graphs. Normal distribution was assessed using the Kolmogorov–Smirnov test. Since none of the variables were normally distributed, the Mann–Whitney *U* Test was applied for comparisons between two groups, and the Kruskal–Wallis Test was used when more than two groups were compared regarding citation counts across different journals. The level of significance was set at *p* < 0.05. Linear regression analysis was conducted, and *R*‐values were calculated to assess the correlation between study quality and citation count.

Two raters, RPV and AMW independently evaluated four scores: MCMS, MINORS, MQCSRE, and BOBQAT. Interrater agreement was measured using the Pearson correlation coefficient, and Cohen's kappa (κ) was interpreted according to the following scale: 0–0.2 represents slight agreement; 0.21–0.4 indicates fair agreement; 0.41–0.6 signifies moderate agreement; 0.61–0.8 shows substantial agreement; and 0.81–1 indicates almost perfect agreement [5].

## RESULTS

### Descriptive statistics

**Figure 2:**
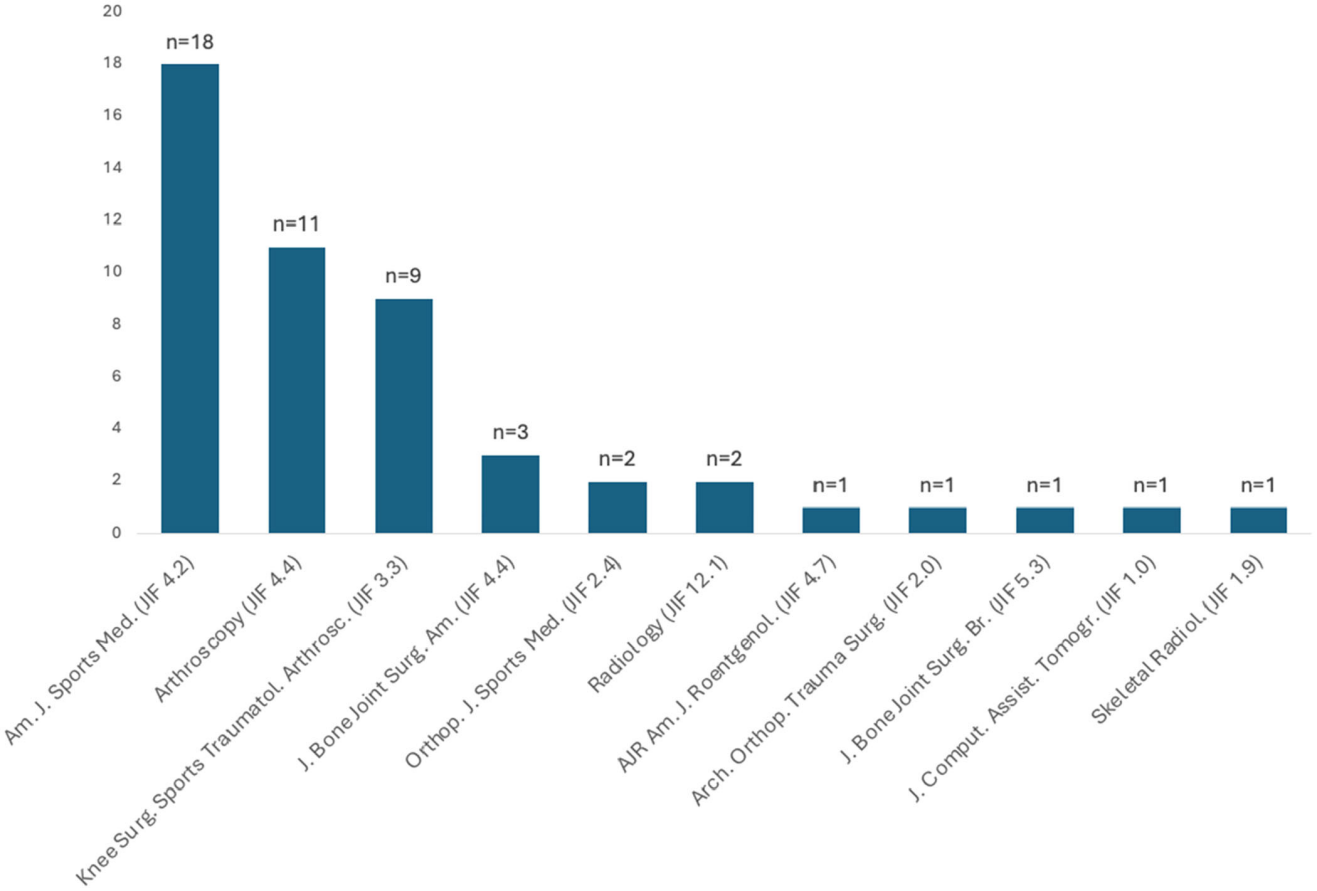
Distribution of the 50 studies by the publication journals and corresponding Journal Impact Factor (JIF) of 2024. Am J Roentgenol, American Journal of Roentgenology; Am J Sports Med, American Journal of Sports Medicine; Arch Orthop Trauma Surg, Archives of Orthopaedic and Trauma Surgery; Arthroscopy, Arthroscopy: The Journal of Arthroscopic & Related Surgery; J Bone Joint Surg Am, Journal of Bone and Joint Surgery, American Volume; J Bone Joint Surg Br, Journal of Bone and Joint Surgery, British Volume; J Comput Assist Tomogr, Journal of Computer Assisted Tomography; Knee Surg Sports Traumatol Arthrosc, Knee Surgery, Sports Traumatology, Arthroscopy; Orthop J Sports Med, Orthopaedic Journal of Sports Medicine; Radiology, Radiology; Skeletal Radiol, Skeletal Radiology.

The 50 most cited studies in the field of meniscus root tears were published between 2004 and 2020. The total citation count was 8234, with a mean of 165 ± 100 citations per article. The most cited study accounted for 721 citations [4], while the least cited study accounted for 92 citations [17]. The top studies were published in 11 different journals, with the *American Journal of Sports Medicine* having the most studies published (*n* = 18; 36%). The second and third most articles were published in the journal *Arthroscopy* (*n* = 11; 22%) and the *Knee Surgery, Sports Traumatology, Arthroscopy* journal (*n* = 9; 18%). The first three journals combined accounted for 38 (76%) of the 50 most cited studies in the field of meniscus root tears (Figure 2).

**Figure 3:**
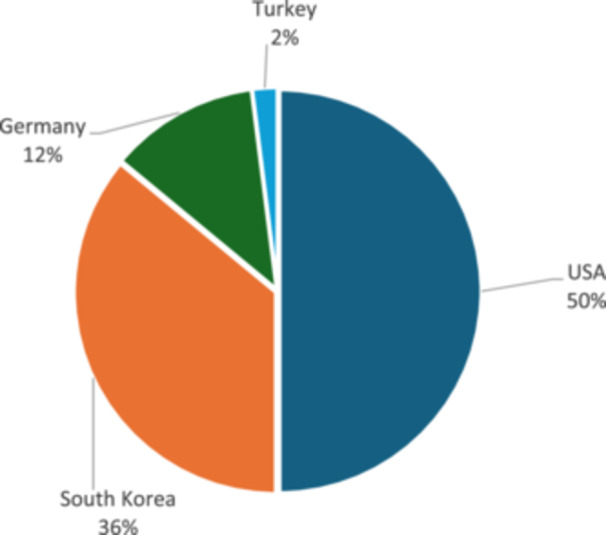
The 50 most cited studies, categorised by the country of the first author's affiliation.

Overall, the 50 most cited studies were published across institutions from four countries, with the United States (*n* = 25; 50%) and South Korea (*n* = 18; 36%) accounting for the largest share (Figure 3).

**Figure 4:**
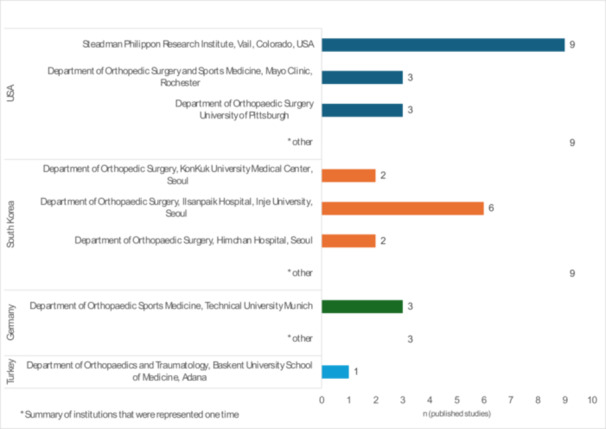
Distribution of the 50 most cited studies on meniscus root tears by institution of publication. Number of studies (*n*).

The earliest published study was in 2004 at the Department of Orthopaedic Surgery, Montefiore Medical Center in Bronx, New York [27]. The study with the highest number of citations (721) was published by the Department of Orthopaedic Surgery at the University of Pittsburgh [4]. The distribution of the 50 most cited studies on meniscus root tears by institutions is shown in Figure 4.

**Figure 5:**
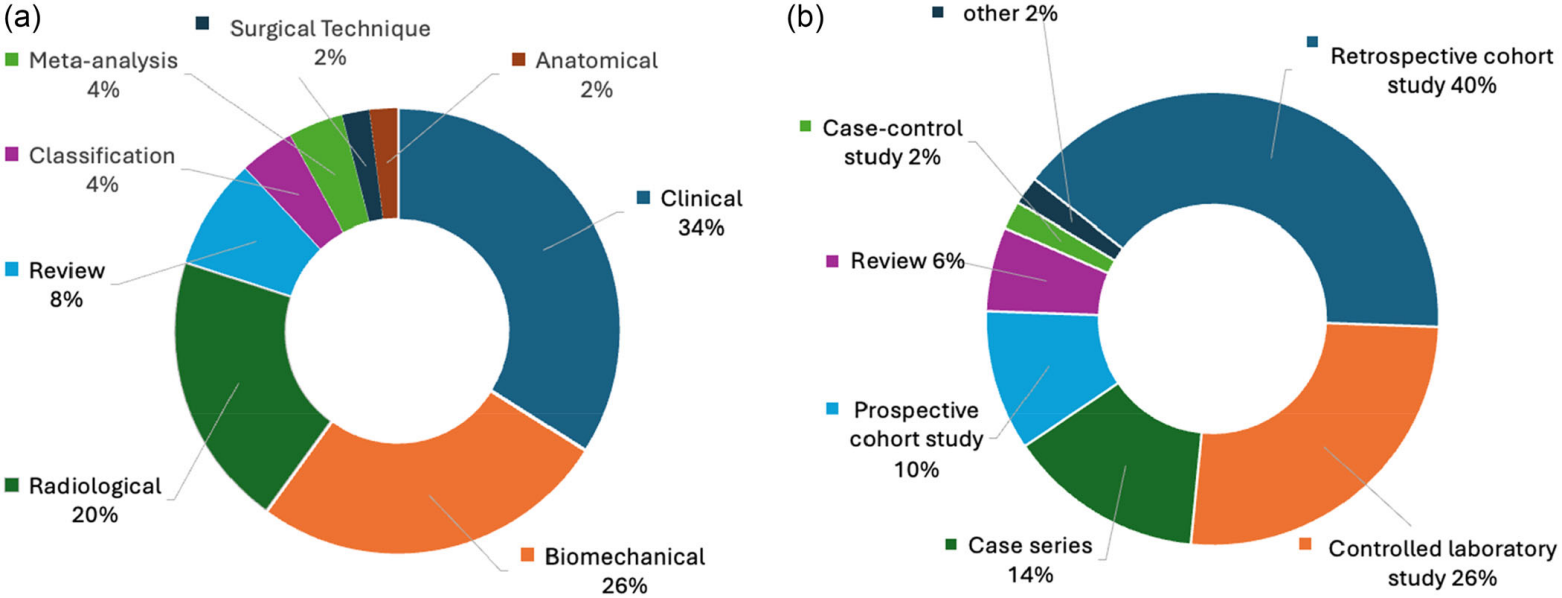
The 50 most cited studies, categorised by their study types (a) and study designs (b).

Considering the different study types, clinical (*n* = 17; 34%) and radiological (*n* = 13; 26%) studies were the most common. A total of eight study types were represented (Figure 5a). Seven different study designs were identified among the 50 most cited studies (Figure 5b). Two‐thirds of the studies consisted of either retrospective cohort studies (*n* = 20; 40%) or controlled laboratory studies (*n* = 13; 26%). A prospective study design was observed in five studies (10%).

### Citation density

The mean citation density, defined as the number of citations per year, among all studies was 15.3 ± 8.1 citations per year. The study with the highest citation density recorded 45.1 citations per year and was published in 2008 [4]. The lowest citation density was 6.4 and the study was also published in 2008 [26].

**Figure 6:**
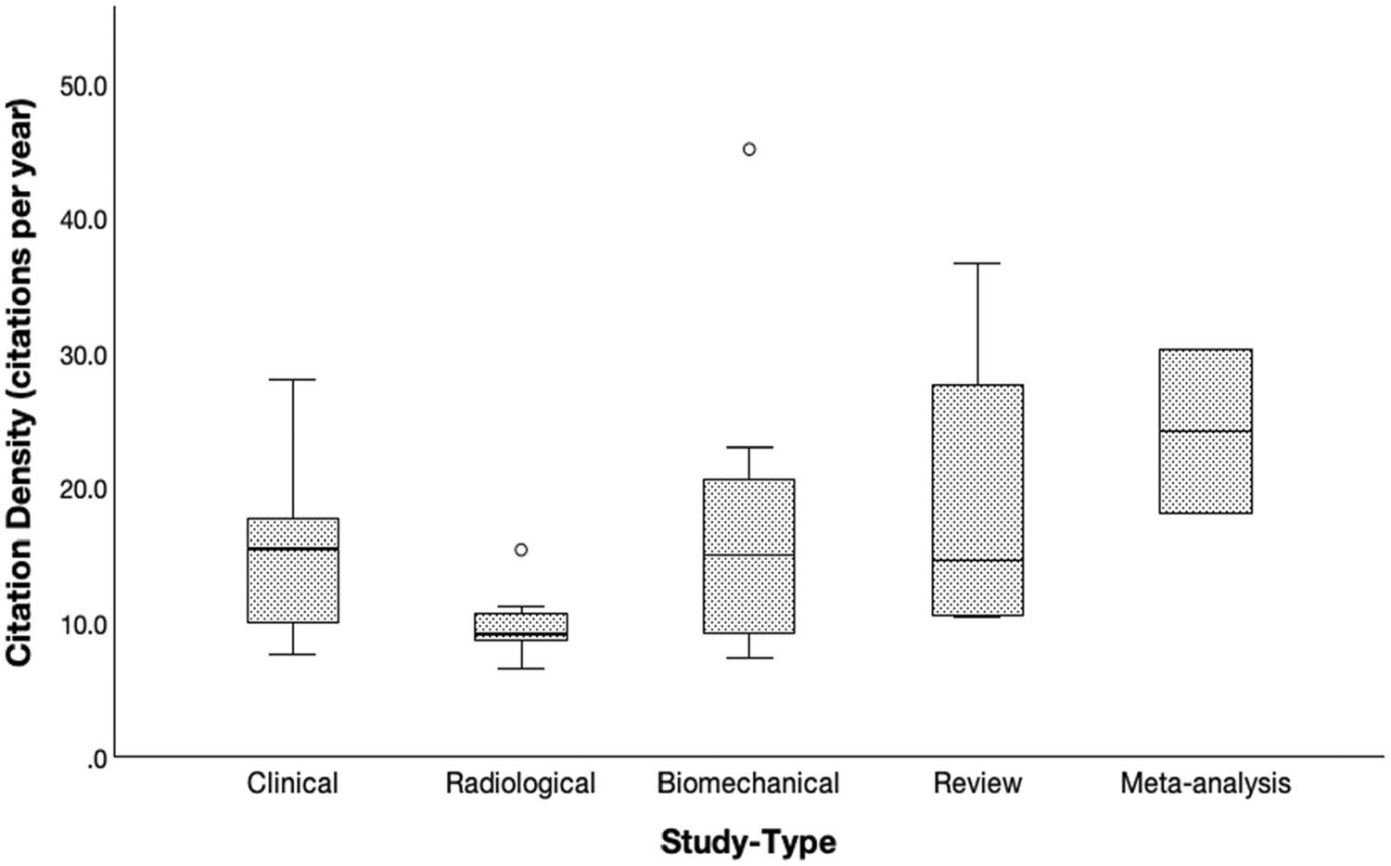
Box plot illustrating the distribution of citation density (citations per year) by study type (Clinical, Radiological, Biomechanical, Review, and Meta‐analysis).

**Figure 7:**
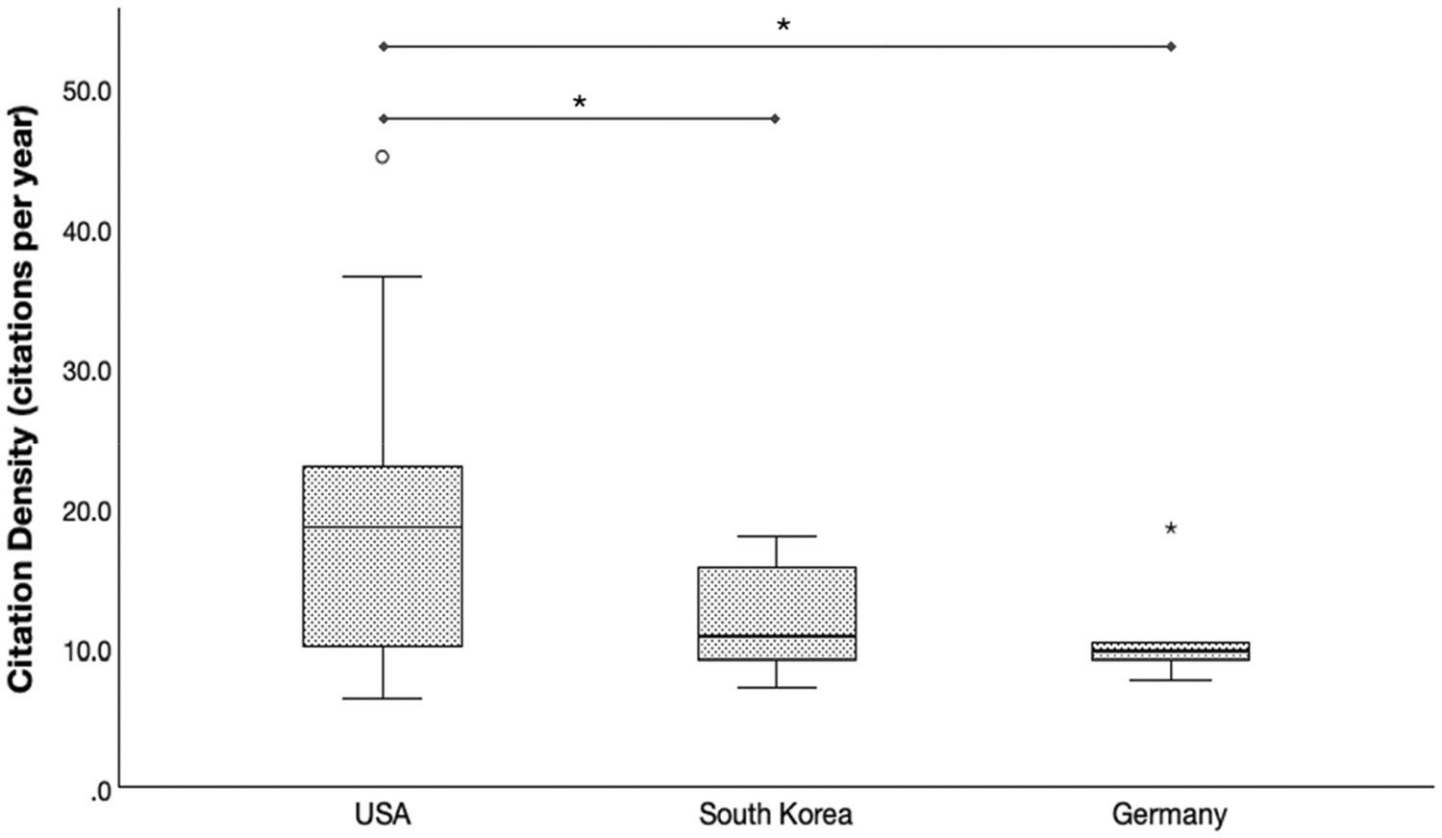
Box plots showing the distribution citation density (citations per year) based on the country of the first author's affiliation. Statistically significant difference (*p < 0.050).

Considering the study type, the difference in mean citation density between clinical and biomechanical studies (17.0 ± 10.1 and 15.0 ± 5.8, respectively) was not statistically significant (*p* = 0.845). However, significant differences between clinical and radiological studies (15.0 ± 5.8 and 9.6 ± 2.5, respectively; *p* = 0.026) and biomechanical and radiological studies (17.0 ± 10.1 and 9.6 ± 2.5, respectively; *p* = 0.022) were observed (Figure 6). Mean citation density was higher in studies published from institutions in the United States (18.7 ± 9.6) compared to institutions from Korea (12.1 ± 3.6; *p* = 0.048) and Germany (10.9 ± 3.9; *p* = 0.040) (Figure 7).

### Study quality

A total of 40 studies (80%) were eligible for methodological scoring. Of the remaining 10 (20%) studies (4 reviews, 2 meta‐analysis, 2 classifications, 1 anatomical and 1 surgical technique) no score was obtained as none was suitable for this purpose. The majority of analysed studies (*n* = 28, 56%) showed a LOE of IV (Table 2). The clinical scores MCMS and MINORS were obtained in 17 studies (34%). Mean MCMS was 64.9 ± 7.0 points (range, 41–73) and mean MINORS was 14.9 ± 4.7 points (range: 12–19). Study quality was assessed for 13 biomechanical studies (46%) with a mean BOBQAT score of 80.6 ± 6.9 points (range, 61–93). The studies that were eligible for the radiological quality evaluation (*n* = 10; 20%) showed a mean MQRSE of 20.2 ± 1.5 points (range, 61–93).

**Table 2:** Distribution of 50 most cited studies in the field of meniscus root tears regarding their Level of Evidence (LOE).

LOE	*n*	(%)
IV	28	56%
III	20	40%
II	1	2%
I	1	2%

**Figure 8:**
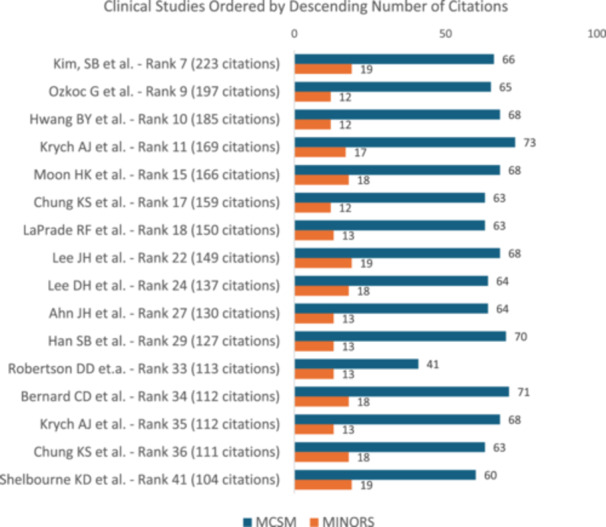
Distribution of the 50 most cited clinical studies and their corresponding quality scores. MCMS, Modified Coleman Methodological Score; MINORS, Methodological Index for Non‐Randomised Studies.

**Figure 9:**
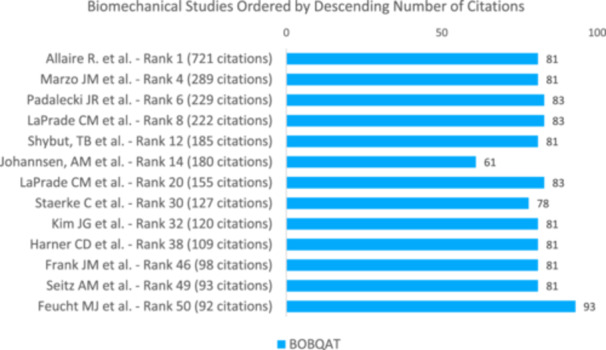
Distribution of the 50 most cited biomechanical studies and their corresponding Biomechanics Objective Basic Science Quality Assessment Tool (BOBQAT) score in the descending order of citations.

Out of all the clinical studies reviewed, three studies (17.6%) scored a MCMS between 70 and 84 points (Figure 7), which is considered 'good' [13]. None of the studies obtained an “excellent” score (85 to 100 points). In total, 8 of all clinical studies (47.1%) received a MINORS score of 16 or higher, which is commonly recognised as the cut‐off for a high‐quality study [32]. When looking at all biomechanical studies, 11 studies (84.6%) scored 81 points or higher (Figure 8), classifying them as good studies [21]. One study (7.7%) achieved a score of 94 points, which is considered to be of excellent methodological quality. Visualisation of the scores of radiological studies is provided in Figure 9.

**Figure 10:**
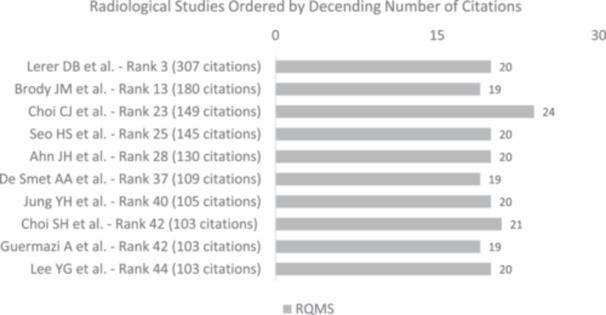
Distribution of the 50 most cited radiological studies and their corresponding Methodological Quality for Clinical Studies of Radiologic Examinations (RQMS) scores in the descending order of citation.

Higher citation count and higher citation densities did not correlate with higher MCMS or MINORS scores in clinical studies (Table 3, Figure 8, *p* > 0.05). Furthermore, a high citation count or high citation density did not correlate with a higher BOBQAT Score in biomechanical studies (Figure 9, *p* > 0.05) or MQRSE scores in radiological studies (Figure 10, *p* > 0.05). A comprehensive list of the 50 most cited studies is provided in Table 4.

**Table 3:** Correlation between total citations and citation density versus methodological quality scores.

		Correlation coefficient *R*	*n*	*p‐*Value
MCMS	vs. citations	0.291	17	0.128
	vs. citation density	0.466		0.060
MINORS	vs. citations	0.085	17	0.137
	vs. citation density	0.223		0.205
BOBQAT	vs. citations	0.048	13	0.438
	vs. citation density	0.009		0.488
MQRSE	vs. citations	0.024	10	0.474
	vs. citation density	0.159		0.331

Abbreviations: BOBQAT, Biomechanics Objective Basic Science Quality Assessment Tool; MCMS, Modified Coleman methodological Score; MINORS, Methodological Index for Non‐Randomised Studies; RQMS, Radiologic Methodology and Quality Scale.

**Table 4:** Comprehensive list of the 50 most cited studies in the field of meniscus root by the journals of publication and their methodological quality assessments, including the Biomechanics Objective Basic Science Quality Assessment Tool (BOBQAT), the scale of methodological quality for clinical studies of radiologic examinations (MQCSRE), the Modified Coleman Methodological Score (MCMS), and the Methodological Index for Non‐Randomised Studies (MINORS).

Rank	Study	Citations	Citation density	Study type	LOE	MINORS	MCMS	MQRSE	BOBQAT
1	Allaire (2008), *J. Bone Joint Surg. Am.* Biomechanical consequences of a tear of the posterior root of the medial meniscus	721	45,1	Biomechanical	4	N/A	N/A	N/A	81
2	Bhatia (2014), *Am. J. Sports Med.* Meniscal root tears significance, diagnosis, and treatment	365	36,5	Review	3	N/A	N/A	N/A	N/A
3	Lerer (2004), *Skeletal Radiol.* The role of meniscal root pathology and radial meniscal tear in medial meniscal extrusion	307	15,4	Radiological	3	N/A	N/A	20	N/A
4	Marzo (2009), *Am. J. Sports Med.* Effects of medial meniscus posterior horn avulsion and repair on tibiofemoral contact area and peak contact pressure with clinical implications	289	19,3	Biomechanical	4	N/A	N/A	N/A	81
5	LaPrade (2015), *Am. J. Sports Med*. Meniscal root tears a classification system based on tear morphology	255	28,3	Classification	4	N/A	N/A	N/A	N/A
6	Padalecki (2014), *Am. J. Sports Med.* Biomechanical consequences of a complete radial tear adjacent to the medial meniscus posterior root attachment site in situ pull‐out repair restores derangement of joint mechanics	229	22,9	Biomechanical	4	N/A	N/A	N/A	83
7	Kim (2011), *Arthroscopy* Medial meniscus root tear refixation: comparison of clinical, radiologic, and arthroscopic findings with medial meniscectomy	223	17,2	Clinical	3	19	66	N/A	N/A
8	LaPrade (2014), *J. Bone Joint Surg. Am.* Altered tibiofemoral contact mechanics due to lateral meniscus posterior horn root avulsions and radial tears can be restored with in situ pull‐out suture repairs	222	22,2	Biomechanical	4	N/A	N/A	N/A	83
9	Kim (2011), *Arthroscopy* Arthroscopic suture anchor repair versus pullout suture repair in posterior root tear of the medial meniscus: a prospective comparison study	204	15,7	Clinical	4	0	65	N/A	N/A
10	Ozkoc (2008), *Knee Surg. Sports Traumatol. Arthrosc.* Radial tears in the root of the posterior horn of the medial meniscus	197	12,3	Clinical	4	12	68	N/A	N/A
11	Hwang (2012), *Am. J. Sports Med.* Risk factors for medial meniscus posterior root tear	185	15,4	Clinical	3	17	73	N/A	N/A
12	Shybut (2015), *Am. J. Sports Med.* Effect of lateral meniscal root tear on the stability of the anterior cruciate ligament‐deficient knee	185	20,6	Biomechanical	4	N/A	N/A	N/A	81
13	Brody (2006), *Radiology* Lateral meniscus root tear and meniscus extrusion with anterior cruciate ligament tear	180	10,0	Radiological	4	N/A	N/A	19	N/A
14	Johannsen (2012), *Am. J. Sports Med*. Qualitative and quantitative anatomic analysis of the posterior root attachments of the medial and lateral menisci	180	15,0	Biomechanical	4	N/A	N/A	N/A	61
15	Krych (2017), *Knee Surg. Sports Traumatol. Arthrosc.* Non‐operative management of medial meniscus posterior horn root tears is associated with worsening arthritis and poor clinical outcome at 5‐year follow‐up	169	24,1	Clinical	3	18	68	N/A	N/A
16	Feucht (2015), *Arthroscopy* Arthroscopic transtibial pullout repair for posterior medial meniscus root tears: a systematic review of clinical, radiographic, and second‐look arthroscopic results	167	18,6	Review	3	N/A	N/A	N/A	N/A
17	Moon (2012), Am. *J. Sports Med.* Prognostic factors of arthroscopic pull‐out repair for a posterior root tear of the medial meniscus	166	13,8	Clinical	4	12	63	N/A	N/A
18	Chung (2015), *Arthroscopy* Comparison of clinical and radiologic results between partial meniscectomy and refixation of medial meniscus posterior root tears: a minimum 5‐year follow‐up	159	17,7	Clinical	3	13	63	N/A	N/A
19	Koenig (2009), *Arthroscopy* Meniscal root tears: diagnosis and treatment	159	10,6	Review	4	N/A	N/A	N/A	N/A
20	LaPrade (2015), *Am. J. Sports Med*. Biomechanical consequences of a nonanatomic posterior medial meniscal root repair	155	17,2	Biomechanical	4	N/A	N/A	N/A	83
21	Faucett (2019), *Am. J. Sports Med.* Meniscus root repair vs meniscectomy or nonoperative management to prevent knee osteoarthritis after medial meniscus root tears: clinical and economic effectiveness	151	30,2	Meta‐Analysis	1	N/A	N/A	N/A	N/A
22	LaPrade (2017), *Am. J. Sports Med*. Posterior meniscal root repairs: outcomes of an anatomic transtibial pull‐out technique	150	21,4	Clinical	3	19	68	N/A	N/A
24	Lee (2009), *Arthroscopy* Arthroscopic pullout suture repair of posterior root tear of the medial meniscus: radiographic and clinical results with a 2‐year follow‐up	149	9,9	Clinical	4	18	64	N/A	N/A
23	Choi (2010), *Arthroscopy* Magnetic resonance imaging evidence of meniscal extrusion in medial meniscus posterior root tear	149	10,6	Radiological	4	N/A	N/A	24	N/A
25	Seo (2011), *Am. J. Sports Med.* Second‐look arthroscopic findings after repairs of posterior root tears of the medial meniscus	145	11,2	Radiological	4	N/A	N/A	20	N/A
26	Chung (2016), *Knee Surg. Sports Traumatol. Arthrosc.* A meta‐analysis of clinical and radiographic outcomes of posterior horn medial meniscus root repairs	144	18,0	Meta‐Analysis	2	N/A	N/A	N/A	N/A
27	Lee (2011), *Knee Surg. Sports Traumatol.* Arthrosc. Predictors of degenerative medial meniscus extrusion: radial component and knee osteoarthritis	137	10,5	Clinical	4	13	64	N/A	N/A
28	Ahn (2010), *Arthroscopy* Results of arthroscopic all‐inside repair for lateral meniscus root tear in patients undergoing concomitant anterior cruciate ligament reconstruction	130	9,3	Clinical	4	18	68	20	N/A
29	Han (2010), *Arthroscopy* Unfavourable results of partial meniscectomy for complete posterior medial meniscus root tear with early osteoarthritis: a 5‐to 8‐year follow‐up study	127	9,1	Clinical	4	13	70	N/A	N/A
30	Staerke (2010), *Arthroscopy* The effect of a nonanatomic repair of the meniscal horn attachment on meniscal tension: a biomechanical study	127	9,1	Biomechanical	4	N/A	N/A	N/A	78
31	Ahn (2007), *Knee Surg. Sports Traumatol. Arthrosc.* A pull out suture for transection of the posterior horn of the medial meniscus: using a posterior trans‐septal portal	121	7,1	Surgical Technique	3	N/A	N/A	N/A	N/A
32	Kim (2013), *Knee Surg. Sports Traumatol. Arthrosc*. Tibiofemoral contact mechanics following posterior root of medial meniscus tear, repair, meniscectomy, and allograft transplantation	120	10,9	Biomechanical	3	N/A	N/A	N/A	81
33	Robertson (2009), *J. Bone Joint Surg. Br.* Meniscal root injury and spontaneous osteonecrosis of the knee an observation	113	7,5	Clinical	4	13	41	N/A	N/A
34	Bernard (2020), *Am. J. Sports Med.* *Medial meniscus posterior root tear treatment: a matched cohort comparison of nonoperative management, partial meniscectomy, and repair*	112	28,0	Clinical	3	18	71	N/A	N/A
35	Krych (2018), Knee Surg. Sports Traumatol. Arthrosc. *Partial meniscectomy provides no benefit for symptomatic degenerative medial meniscus posterior root tears*	112	18,7	Clinical	3	13	68	N/A	N/A
36	Chung (2017), Am. J. Sports Med. *Pullout Fixation of Posterior Medial Meniscus Root Tears: Correlation Between Meniscus Extrusion and Midterm Clinical Results*	111	15,9	Clinical	3	18	63	N/A	N/A
37	De Smet (2009), AJR Am. J. Roentgenol. *MR Diagnosis of Posterior Root Tears of the Lateral Meniscus Using Arthroscopy as the Reference Standard*	109	7,3	Radiological	4	N/A	N/A	19	N/A
38	Harner (2009), J. Bone Joint Surg. Am. *Biomechanical Consequences of a Tear of the Posterior Root of the Medial Meniscus Surgical Technique*	109	7,3	Biomechanical	4	N/A	N/A	N/A	81
39	DePhillipo (2019), Am. J. Sports Med. *Quantitative and Qualitative Assessment of the Posterior Medial Meniscus Anatomy: Defining Meniscal Ramp Lesions*	108	21,6	Anatomical	3	N/A	N/A	N/A	N/A
40	Jung (2012), Am. J. Sports Med. *All‐Inside Repair for a Root Tear of the Medial Meniscus Using a Suture Anchor*	105	8,8	Radiological	4	N/A	N/A	20	N/A
41	Shelbourne (2011), Am. J. Sports Med. LoNg‐term Evaluation Of Posterior Lateral Meniscus Root Tears Left In Situ At The Time Of Anterior Cruciate Ligament Reconstruction	104	8,0	Clinical	3	19	60	N/A	N/A
42	Choi (2012), Knee *Surg. Sports Traumatol. Arthrosc.* The MRI findings of meniscal root tear of the medial meniscus: emphasis on coronal, sagittal and axial images	103	8,6	Radiological	3	N/A	N/A	21	N/A
43	Guermazi (2013), *Radiology* Medial posterior meniscal root tears are associated with development or worsening of medial tibiofemoral cartilage damage: the multicenter osteoarthritis study	103	9,4	Radiological	3	N/A	N/A	19	N/A
44	Lee (2008), *J. Comput. Assist. Tomogr.* Magnetic resonance Imaging findings of surgically proven medial meniscus root tear: Tear configuration and associated knee abnormalities	103	6,4	Radiological	3	N/A	N/A	20	N/A
45	Petersen (2014), *Arch. Orthop. Trauma Surg.* Posterior root tear of the medial and lateral meniscus	103	10,3	Review	4	N/A	N/A	N/A	N/A
46	Frank (2017), *Orthop. J. Sports Med.* Lateral meniscus posterior root and meniscofemoral ligaments as stabilising structures in the acl‐deficient knee: a biomechanical study	98	14,0	Biomechanical	4	N/A	N/A	N/A	81
47	Sung (2013), *Arthroscopy* Meniscal extrusion and spontaneous osteonecrosis with root tear of medial meniscus: comparison with horizontal tear	96	8,7	Radiological	3	N/A	N/A	N/A	N/A
49	Seitz (2012), *Orthop. J. Sports Med.* Effect of partial meniscectomy at the medial posterior horn on tibiofemoral contact mechanics and meniscal hoop strains in human knees	93	7,8	Biomechanical	4	N/A	N/A	N/A	81
48	Forkel (2015), *Knee Surg. Sports Traumatol. Arthrosc.* Different patterns of lateral meniscus root tears in ACL injuries: application of a differentiated classification system	93	10,3	Classification	3	N/A	N/A	N/A	N/A
50	Feucht (2014), *Am. J. Sports Med.* Biomechanical comparison between suture anchor and transtibial pull‐out repair for posterior medial meniscus root tears	92	9,2	Biomechanical	4	N/A	N/A	N/A	93

Abbreviations: AJR Am J Roentgenol, American Journal of Roentgenology; Am J Sports Med, American Journal of Sports Medicine; Arch Orthop Trauma Surg, Archives of Orthopaedic and Trauma Surgery; Arthroscopy, Arthroscopy: The Journal of Arthroscopic & Related Surgery; J Bone Joint Surg Am, Journal of Bone and Joint Surgery, American Volume; J Bone Joint Surg Br, Journal of Bone and Joint Surgery, British Volume; J Comput Assist Tomogr, Journal of Computer Assisted Tomography; Knee Surg Sports Traumatol Arthrosc, Knee Surgery, Sports Traumatology, Arthroscopy; Orthop J Sports Med, Orthopaedic Journal of Sports Medicine; Radiology, Radiology; Skeletal Radiol, Skeletal Radiology.

Interrater analyses showed substantial agreement for the MCMS (*κ* = 0.77), MINORS (*κ* = 0.64), and BOBQAT (*κ* = 0.61) and almost perfect agreement for the MQCSRE (*κ* = 0.89) between the two raters (11).

## DISCUSSION

The most important finding of this study was that objective quality scores did not correlate with higher citations nor citation densities in the 50 most cited studies on meniscus root tears. Overall, study quality according to the quality scores was moderate, the most common study type was clinical studies, and the most common study design was retrospective cohort studies. The majority of all studies were published in the *American Journal of Sports Medicine* and the *Arthroscopy* Journal.

The study with the highest number of citations evaluated the biomechanical effect on tibiofemoral cartilage in knee joints with meniscus root tears and investigated the restoration of contact pressures after meniscus root repair [4]. It was conducted at the Department of Orthopaedic Surgery of the University of Pittsburgh. This study provided the biomechanical rationale for treating this condition, as evidenced by its 721 citations. The BOBQAT score was 81 which represents a good study quality [21]. The study did not describe bone density measurements nor cyclic loading, which explains the BOBQAT score deduction. The second most cited study systematically reviewed the anatomy of meniscus root tears, diagnosis, and biomechanics and furthermore provided a treatment algorithm [9]. One reason for the high number of citations of 365 may be its publication year of 2014. Since then, the study has shown a citation density of 37 times per year, which represents the second‐highest citation density among the 50 most cited studies in publications on meniscus root tears. No score was obtained for this study due to its study design. The third most cited study showed that medial meniscus extrusion ≥ 3 mm was strongly associated with degenerative joint disease and medial meniscus radial or root tear [27]. The study obtained a MQRSE score of 20 out of 30 points, which represents the median of all radiological studies. The study did not compare its findings to a reference standard, and the measurement reliability was only briefly described, which resulted in a decreased score. The most frequently cited clinical study (*n* = 223, rank 7) showed that arthroscopic pullout repair was superior to partial meniscectomy in the case of medial meniscus root tears [24]. The study received a MINORS score of 19, which is above the mean (14.9) compared to the remaining clinical trials and states high study quality [32]. The study obtained an MCMS of 66 points, which is just above the mean value of the other clinical studies and states moderate study quality [13] (64.9). Only three clinical studies obtained a score between 70 and 84 points and were considered to achieve good study quality. No clinical study was able to achieve an excellent score ( > 84 points). The reason for this lies in the MCMS's judgement of the group size, the follow‐up period, and above all, in the assessment of the study design (0 points for retrospective studies) [13]. These studies represent the biomechanical, radiological, and therapeutic principles underlying the successful treatment of meniscus root tears according to the latest knowledge.

Almost 9 out of 10 studies originated from the United States or South Korea, showing a condensation of competence in certain centres to treat meniscus root tears. Traditionally, there is a dominant proportion of studies published in the United States with similar bibliometric studies, and it is not unique to orthopaedic specialties [1, 3, 16, 28, 29, 36]. The Steadman Philippon Research Institute in Vail, the Department of Orthopaedic Surgery and Sports Medicine at Mayo Clinic in Rochester, and the Department of Orthopaedic Surgery at the University of Pittsburgh contributed 15 of the 50 most cited studies. Notably, 36% of all published studies on meniscus root tears were written by authors with an institutional affiliation in South Korea. Of them, the Department of Orthopaedic Surgery, Ilsanpaik hospital of the Inje University in Seoul published six studies. This underlines the occurrence of competence in this field of research. The reason for this may be an overall higher incidence of posterior meniscus root tears in the Asian population, which was suggested to be due to the higher incidence of constitutional varus and cultural reasons [10, 37]. Between 2014 and 2015, three studies were published by authors from the Department of Orthopaedic Sports Medicine at the Technical University of Munich. Notably, the biomechanical study that received the highest BOBQAT score (93 points) was included among them [17].

Most articles analysed in this study (76%) were published in the *American Journal of Sports Medicine* (JIF of 2024: 4.2), The *Journal of Arthroscopic & Related Surgery* (JIF of 2024: 4.4) and the *Journal of Knee Surgery, Sports Traumatology, Arthroscopy* (JIF of 2024: 3.2). These journals are ranked among the top journals in the sports‐orthopaedic field according to the Clarivate™ (Web of Science™, all rights reserved). Out of a total of 8234 citations from the 50 list, 5973 citations came from studies published in these three journals. This highlights the significance interest among experts in the field of meniscus root tears.

Bibliometric studies highlight global trends and interest in meniscus root tears by examining the frequency of citations accrued by individual articles. When interpreting bibliometric data, it is essential to consider certain biases. For example, the 'snowball effect' describes a phenomenon where heavily cited articles are more likely to be referenced in subsequent research, amplifying their visibility and impact. Similarly, articles published early in a new research area often become foundational and are cited more frequently as the field develops. Another reason for a higher visibility of a study may be the impact of one of the authors in the expert community. Articles written by influential figures may tend to be cited more frequently [25]. The number of citations an article receives depends on several factors, including the quality of the work, the relevance of its topic, the time since publication, and its contribution to advancing research.

Earlier published studies tend to receive more citations on average than more recent work, as demonstrated by this article. Citation density serves as a straightforward tool to contextualise total citations over time. In contrast, the so‐called 'obliteration by incorporation' effect can lead to an underestimation of total citations. This effect refers to the inclusion of previously accepted knowledge in newer research, resulting in fewer citations for older studies, in particular [19]. In addition, articles published before the introduction of the Internet could be even more strongly affected by this effect.

This study analysed the 50 most cited studies in the field of meniscus root tears and found that 48 of them were Level III studies or lower. This underlines the need for more high‐level studies (LOE I and II) in this field of research, which has also been emphasised in a recent systematic review [18]. However, this is not a unique phenomenon compared to other fields of interest in the orthopaedic spectrum. The specialty's given nature is why Level I and II studies are more difficult to perform. Due to this, it is even more important to keep high methodological quality.

This study has several limitations. Objective quality assessment of studies is very important to give a brief estimate of the methodological strength of a study. A randomised controlled trial that is triple‐blinded is the highest quality standard of interventional or clinical studies. It lies in the nature of the surgical specialties, that this standard is not always implementable and therefore, the objective quality tools may underscore certain studies. The scores in this study were designed to objectivise the methodology of a certain study type. The MCMS favours prospective cohort studies, and the MINORS emphasises studies with a long follow‐up time and detailed rehab protocol [13, 32]. The subjectivity of individual scores may introduce bias in the grading of certain articles. Only five of the studies employed a prospective design, while more than half (56%) were retrospective studies. Despite their lower scores for study design, these retrospective studies performed well in other evaluation categories. As a result, the MCMS or MINORS Score may not accurately reflect the true quality of the studies, but it makes them comparable on an objective basis. Recently, the BOBQAT score was published to provide a quality assessment for biomechanical studies. In the current analysis, 13 studies were identified as purely biomechanical, allowing for a biomechanical quality score to be established for comparison among these studies. Similarly, all ten radiological studies were evaluated by a purely radiological quality score, the MQRSE. Consequently, comparability between the different scores of different study designs is limited. It was not possible to determine a quality score for every study. Additionally, there were a total of 10 studies for which no quality scores could be calculated due to their study design. In comparison to previously published bibliometric studies, the number of studies lacking a score is relatively low [1, 35]. Unknown effects may arise from the dynamics and mechanisms of scientific writing. Highly cited papers are more likely to demonstrate significant intellectual influence, which can change readers' perceptions of quality. Papers with low citation counts may be perceived as being of lower quality. It is difficult to numerically capture and objectively estimate this effect [25, 34]. Additionally, earlier published studies tend to receive more citations on average than more recent work, as demonstrated by this article. Citation density serves as a straightforward tool to contextualise absolute citation counts over time. In contrast, the so‐called “obliteration by incorporation” effect can lead to an underestimation of citation counts. This effect refers to the inclusion of previously accepted knowledge in newer research, resulting in lower citation counts for older studies, in particular [19].

## CONCLUSION

The methodological quality scores of the 50 most cited studies on meniscus root tears were moderate. Furthermore, the number of citations did not correlate with these quality scores, and the most frequently cited studies did not have the highest quality scores. There is a need for more level I and II studies in the field of research on meniscus root tears. This list can serve as a valuable reference for orthopaedic surgeons seeking to explore the literature on meniscus root tears.

## AUTHOR CONTRIBUTIONS

Romed P. Vieider and Volker Musahl designed the study. Anja M. Wackerle, Romed P. Vieider collected the data and performed the measurements. Romed P. Vieider and performed the statistical analysis. Romed P. Vieider, Karina Dias, Anja M. Wackerle and Camila Grandberg wrote the manuscript. Volker Musahl, Jonathan D. Hughes and Bryson P. Lesniak assisted with data interpretation and critically reviewed the manuscript. All authors read and approved the final manuscript.

## CONFLICT OF INTEREST STATEMENT

Volker Musah reports educational grants, consulting fees and speaking fees from Smith & Nephew plc, educational grants from Arthrex and DePuy/Synthes, is a board member of the International Society of Arthroscopy, Knee Surgery and Orthopaedic Sports Medicine (ISAKOS), and deputy editor‐in‐chief of Knee Surgery, Sports Traumatology, Arthroscopy (KSSTA). Bryson P. Lesniak received payment from Mid‐Atlantic Surgical Systems for education. Jonathan D. Hughes has received grant support from Arthrex, education payments from Mid‐Atlantic Surgical Systems and Smith + Nephew, and hospitality payments from SI‐BONE and Stryker and is on the editorial board of Knee Surgery, Sports Traumatology, Arthroscopy (KSSTA). Volker Musahl received consulting fees from Smith & Nephew and Newclip, educational fees from Arthrex, DePuy Synthesis, and Conmed, and is a board member of the International Society of Arthroscopy, Knee Surgery and Orthopaedic Sports Medicine (ISAKOS), and the Assistant editor‐in‐chief of KSSTA. Romed Peter Vieider, Mathew Kolevar, Karina Dias, Camila Grandberg and Anja Maximiliane Wackerle do not declare any conflict of interest.

## ETHICS STATEMENT

For this study, no ethics approval was required.

## Data Availability

The data that support the findings of this study are available from the corresponding author upon reasonable request.
